# Human immunodeficiency virus, syphilis and hepatitis C virus prevalence trends among cross-border migrant Vietnamese female sex workers in Guangxi, China

**DOI:** 10.1186/s12889-015-2561-0

**Published:** 2015-12-09

**Authors:** Chen Zhang, Xiaoming Li, Yu Liu, Shan Qiao, Yuejiao Zhou, Zhenzhu Tang, Zhiyong Shen

**Affiliations:** Department of Epidemiology, Vanderbilt University, 2525 West End Ave. Suite 725, Nashville, TN 37203 USA; University of South Carolina, Arnold School of Public Health, 915 Greene Street, Room 408, Columbia, SC 29208 USA; Department of HIV/STD Prevention, Guangxi Center for Disease Control and Prevention, Jinzhou Rd., Qingxiu District, Nanning, Guangxi China; Guangxi Center for Disease Control and Prevention, Jinzhou Rd., Qingxiu District, Nanning, Guangxi China

## Abstract

**Background:**

Global literature indicates the burden of human immunodeficiency virus (HIV), syphilis and hepatitis C virus (HCV) has disproportionately affected cross-border migrant female sex workers (FSW). However, few studies reported the HIV risk among Vietnamese FSW at borderline areas in China. We examined five consecutive years of HIV, syphilis, and HCV prevalence and corresponding risk factors among this group in Guangxi Province of China in the current study.

**Method:**

Demographic and behavioral data as well as test results of blood samples for HIV/syphilis/HCV testing were collected from the annual National Sentinel Surveillance (NSS) from the year of 2010 to 2014. The prevalence trends were first examined by stratified demographic and behavioral status. Predictive models with logistic regression were further employed to identify risk predictors for HIV, syphilis and HCV combined with multiple imputation for missing data as well as restricted cubic splines for key continuous covariates. Moreover, weighted prevalence using the distribution of venue types among all FSW from the NSS survey as the standardized population was also reported.

**Results:**

The overall prevalence of HIV, syphilis and HCV across the five year period was 3.2 % (95 % CI = 2.1 %,4.3 %), 6.9 % (95 % CI = 5.3 %,8. %), and 2.6 % (95 % CI = 1.6 %,3.6 %), respectively. HIV prevalence changed from 8.2 % (95 % CI = 0.5 %,15.9 %) in 2010 to 1.7 % (95 % CI = 0.4 %,3.0 %) in 2014, and the prevalence decreased notably among FSW who were younger than 25 years old, stayed less than six months, and who participated in the HIV prevention services (*P* < 0.05). The syphilis prevalence also ranged from 8.2 % (95 % CI = 0.5 %,15.9 %) in 2010 to 3.9 % (95 % CI = 1.9 %,5.9 %) in 2014, and the prevalence remained relatively stable among FSW who reported inconsistent condom use with clients in the past month, those who did not participate in HIV prevention services, and had lower HIV knowledge (*P* > 0.05). HCV prevalence increased from 0 % in 2010 to 2.2 % (95 % CI = 0.7 %, 3.7 %) in 2014. Multivariable analyses revealed that infection with HCV increased the odds of HIV and syphilis infection. Drug use (aOR = 44.0, 95C % = 16.3,129.5) increased the odds of HCV infection.

**Conclusions:**

The relatively higher HIV, syphilis and HCV prevalence among Vietnamese FSW compared to their Chinese counterparts sets a challenge for health officials at both sides. To curb the epidemic among the cross-border FSW, preventive action requires bilateral cooperation and action by health authorities of China and Vietnam. A national-level response system should be launched in order to tackle the urgently ever-increasing epidemic.

## Background

Compared to Western and African countries, the HIV epidemic in Asian countries started a decade later. In the early to mid-1980s, while other parts of the world began to tackle the severe HIV epidemic, Asia remained unaffected until the early 1990s. To date, there is an estimated five million People Living with HIV and AIDS (PLWHA) in this continent [1]. Similarly, the great burden of other sexually transmitted diseases (STD) including hepatitis C virus (HCV) and syphilis infection was also observed [2]. Unprotected heterosexual sex is one of the major transmission routes in Asia, and female sex workers (FSW) play a significant role in HIV as well as other STD transmission [3–5]. Compared with FSW in other continents, FSW in Asia bear the highest burden of infection of HIV and other STD [6].

Global literature has documented a high risk among migrant sex workers [7]. For instance, for Nepalese FSW, the HIV prevalence among Indian returnees was 3–6 times higher than their non-migrant local counterparts [7]. In another study conducted in Yunnan of China, the HIV prevalence among 600 Burmese migrant women was as high as 2.17 % [8]. High prevalence of STD (e.g., syphilis, gonorrhea, and chlamydia) has been reported in a study conducted in five border provinces of Vietnam [9]. Evidence suggests that the synergistic effect of disempowerment, social marginalization, and high mobility provides fertile ground for the rapid transmission of HIV among this vulnerable group [10, 11]. The disempowered status is a consequence of the following factors: lack of legal documentation, desperation for quick financial returns, inability to speak the local language and having no local connections [7]. Therefore, they heavily rely on gatekeepers/managers of the brothels where they stay for their livelihood, protection and financial survival [11, 12]. As a result, they don’t even have the power to decline any violent clients or negotiate for safe sex if their clients insist on not using condoms during sex [11]. Furthermore, their illegal migration status or non-citizenship makes them socially marginalized and excluded from preventative services and medical treatment. For instance, in Thailand, migrant FSW are not eligible for the country-specific HIV prevention programs and are forced to seek for help from physicians privately [10]. Even for programs targeting migrant FSW, the high mobility renders them invisible and unreachable by existing outreach and education programs that provide HIV risk reduction strategies [13]. In addition, pervasive ethnic discrimination in the destination country often excludes them from actively seeking for assistance [14].

In China, approximately 4–10 million FSW are working in the sex industry [15]. Among these FSW, a small portion of them come from other countries and engage in sex work in economically developed cities or borderline regions [14]. As a major composition of the work force of the thriving sex industry in borderline regions of China, Vietnamese women are noteworthy. A study conducted in a China-Vietnam border county with mixed FSW population depicted the intimidating epidemic, with 58.3 % infected with HSV-2, 5.5 % infected with HIV, and 0.6 % infected with syphilis [16]. Our previous study also identified a high HIV prevalence of 8.6 % among a small portion of Vietnamese FSW in China, among whom 5.6 % co-infected with HCV and one-fourth co-infected with syphilis [17]. In the meantime, it is suggested that the injecting drug users (IDU) concentrated epidemic has transited to sex-driven epidemic in China-Vietnam border regions [18]. The high prevalence of HIV, HCV and syphilis as well as their prevailing risk behaviors among this cross-border FSW group brought a great concern to health officials as well as research community in both China and Vietnam. Yet, no serological and behavioral data have been published among this at-risk population.

An active National Sentinel Surveillance (NSS) system has been established to monitor HIV as well as HCV and syphilis among high-risk populations (e.g., FSW, injected drug users) in China since mid-1990s [19, 20]. Based upon the NSS protocol, a diagnosis procedure included two sequential steps: an initial screening test (e.g., a test for antibody of the pathogen) was implemented among all participants, and a confirmative test (e.g., a test for antigen of the pathogen) would be followed only if the initial test showed a positive result [21, 22]. In the current study, a secondary analysis of the NSS data in Guangxi (2010–2014) was used to assess the epidemic of HIV, HCV and syphilis among Vietnamese FSW working in China. Predictive models were further employed to identify risk factors for the each type of infection among this marginalized population.

## Methods

### Study setting

Guangxi Zhuang Autonomous Region (Guangxi) shares about a 750 kilometers long open border with Vietnam in the South with a population of 46.8 million [23]. The Sino-Vietnam border region is bustling with business and individuals who come from across Dongxing-Mong Cai border region to make a living [24]. Meanwhile, the burgeoning economic has been accompanied by the ever-increasing HIV epidemic in Guangxi, especially in the borderline regions [18]. Based upon an official report, by June 2011, a total of 75,716 HIV/AIDS cases had been reported, which placed Guangxi the second among 31 provinces and districts in China in terms of HIV seropositive cases with a HIV prevalence of 0.16 % [25–27]. The major transmission route in Guangxi is unprotected heterosexual sex [25–27].

### Study design

We performed secondary analysis of the NSS data in the current study. The detailed procedure of data collection has been published elsewhere [17]. In the current study, we investigated a subgroup of cross-border FSW from the overall FSW that were examined in previously published study [17]. Briefly, five cross-sectional surveys were conducted yearly from 2010 through 2014 in 35 sites that cover both urban districts and rural counties in Guangxi. Eligible participants were those who were at least 18 years old; worked in one of the venues/sites (e.g., sauna, public bath center, night club, karaoke, dance hall, bar, hotel, restaurant, hair salon, massage parlor, mini-hotel, road-side restaurant, and street) that were known to local Center for Disease Control and Prevention (CDC) for provision of commercial sex services, and these venues were categorized into high, medium and low-paying venues based upon the local socioeconomic status; provided written consent form and completed self-administered, standardized behavioral surveillance survey; and provided blood samples for HIV, syphilis and HCV testing. For FSW who could not read Chinese, the survey was done in their own language (i.e., Vietnamese) or with the assistance of a Vietnamese-Chinese interpreter. For individuals who tested positive for any of the infections, they were referred to an appropriate local hospital for further evaluation and management. The NSS for FSW in Guangxi covered a total of 1,026 women (49 in 2010, 110 in 2011, 166 in 2012, 343 in 2013, and 358 in 2014) who self-identified as Vietnamese and were included in the current analysis (Table 1). The Institute Review Board at Guangxi CDC in China approved the study protocol.

**Table 1:** Prevalence of HIV, syphilis and HCV among Vietnamese and overall FSW (unweighted vs. weighted)

		2010 (N = 49)	2011 (N = 110)	2012 (N = 166)	2013 (N = 343)	2014 (N = 358)	Overall (N = 1026)
HIV (%)	VFSW-Unweighted	8.16	5.45	3.61	3.21	1.68	3.22
	VFSW-Weighted^1^	5.65	2.95	4.26	3.23	1.53	2.84
	All-FSW	1.60	1.07	1.11	0.88	0.65	1.00
Syphilis (%)	VFSW-Unweighted	8.16	9.09	9.64	7.87	3.91	6.92
	VFSW-Weighted^1^	3.45	6.55	7.13	7.99	3.59	5.92
	All-FSW	6.10	7.24	7.14	6.28	4.68	6.22
HCV (%)	VFSW-Unweighted	0.00	2.73	2.41	3.50	2.23	2.63
	VFSW-Weighted^1^	0.00	1.47	2.51	2.33	2.57	1.67
	All-FSW	1.00	0.96	1.09	0.95	0.73	0.93

Notes: 1. Direct standardization: using the all FSW’s as the standard population for each stratum of venues to obtain expected number of events, summing the events to obtain total expected events and divided by total standard population to obtain standardized risk. The general formula is: *Rw* = ∑^&# x200B;^(*wiRi*)/∑^&# x200B;^(*wi*), where w_i_ is the stratum specific weight of the standard generation; R_i_ is the stratum specific risk of the study population

Questions in the behavioral surveillance survey assessed the following domains: demographics (e.g., age, education, marital status, length of working), behavioral factors (e.g., condom use with clients in the last sex act, condom use with clients in the last month, history of drug use), and other key factor (e.g. HIV knowledge, participation in HIV services). All categorizations were made either based upon previously published literature or the data distribution.

Based upon the NSS protocol, blood samples were collected from all participants for HIV, syphilis and HCV testing as part of the surveillance procedure [22]. For HIV, screening tests were conducted using Enzyme-Linked Immunosorbent Assays (ELISA) method (ELISA-1 for HIV antibody testing) and confirmation tests were done only among positive cases using a different ELISA method (ELISA-2 for HIV antigen testing) with microplate readers. A result was considered positive only if the confirmation test was also positive. The HIV testing kits were produced by Yingke Xinchuang Technology Company, and Beijng Wantai Pharmaceutical Company in China. For HCV, an ELISA for antibody testing was use as screening test. If the test result was positive, a PCR (polymerase chain reaction) test would be used to detect HCV-RNA (ribonucleic acid) for confirmation. The HCV testing kits were produced by Abbott Laboratories in the US. Two different methods (ELISA and RPR [rapid plasma regain]) were used to conduct parallel tests for syphilis and a result was considered positive only if it was positive on both tests. The syphilis testing kits were produced by Beijing Jinhao Pharmaceutical Company in China and Fuji Industrial CO. LTD in Japan, respectively. All the initial screening and confirmation tests were conducted at designated and certified laboratory at local CDC or hospitals. All HIV, syphilis and HCV infections were served as the outcome variables in the following predictive multivariate logistic models.

### Analytical procedure

First, Chi-square test was used to examine the associations of demographic and behavioral factors and the prevalence of HIV, syphilis and HCV by different years. A trend test (test for trend across ordered groups by ‘nptrend’ command in STATA) was used to examine if any of these factors varied over time. In addition, HIV, syphilis and HCV prevalence was also tested within different strata of demographic and behavioral factors as well as the trend changes over time. Furthermore, standardized prevalence among Vietnamese FSW was calculated using the distribution of venue types among all FSW in the Guangxi NSS from 2010–2014 as the standardized population structure.

In the process of building predictive models of identifying risk factors for each specific type of infection (e.g., HIV, syphilis, and HCV), we followed standard protocol of predictive modeling [28, 29]. First, univariate analyses were conducted to find covariates with the significance level of p < 0.20 to make the model more conservative. After identifying potential covariates from the univariate analyses, we employed multivariable logistic regression model to find significant risk factors. We also reported a predictive value for each model, which was measured by the areas under ROC curve (AUC). Based upon empirical criteria, AUC ≥0.80 meant some utility in predicting outcomes of individual subjects by independent variables [30]. In addition, we used the restricted cubic spline (RCS) with three knots to model key continuous covariates (e.g., age) in these models in order to better capture the dose–response relationship and relax the linear assumption [31, 32]. Multiple imputation was employed for handling covariates (e.g., HIV knowledge) with 5-15 % missing values [33]. Data were imputed 50 times using predictive mean matching imputation method to fill in missing values of a continuous variable. All data analyses were performed using STATA software (version 12.0, College Station, TX).

## Results

### Prevalence, demographic and behavioral characteristics

**Fig. 1:**
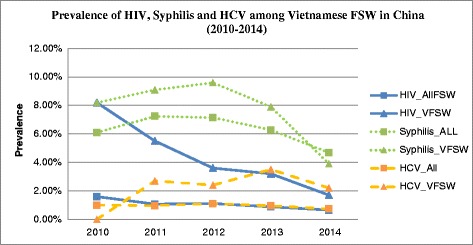
Prevalence of HIV, Syphilis and HCV among Vietnamese FSW in China (2010–2014). The blue solid line represents HIV among all and Vietnamese FSW: The HIV prevalence among all FSW ranged from 1.60 % in 2010 to 0.65 % in 2014; the HIV prevalence among Vietnamese FSW ranged from 8.16 % in 2010 to 1.68 % in 2014. The green dot line represents syphilis among all and Vietnamese FSW: The syphilis prevalence among all FSW ranged from 6.10 % in 2010 to 4.68 % in 2014; the syphilis prevalence among Vietnamese FSW ranged from 8.16 % in 2010 to 3.91 % in 2014. The orange dash line represents HCV among all and Vietnamese FSW: The HCV prevalence among all FSW ranged from 1.00 % in 2010 to 0.73 % in 2014; the HCV prevalence among Vietnamese FSW ranged from 0.00 % in 2010 to 2.23 % in 2014

The overall prevalence of HIV, syphilis and HCV among Vietnamese FSW across the five year was shown in Table 1 and Fig. 1. After weighting with all included FSW in the NSS by their venue types in each specific year, the overall prevalence for these three infections dropped to from 3.2 % to 2.8 % for HIV, 6.9 % to 5.9 % for syphilis, and 2.6 % to 1.7 % for HCV, respectively. We also found both crude and weighted prevalence of HIV, syphilis and HCV among Vietnamese FSW was higher compared to all FSW in the NSS survey (Table 1; Fig. 1).

Some statistically significant differences in demographic and behavioral characteristics (e.g., proportions) were observed over the five-year period. For instance, the proportion of being married and using condoms in the last sex as well as in the last month got increased over the five-year period (*P* for trend <0.05, Table 2). Most Vietnamese FSW reported being married, having used a condom use in the last sex act, and reported consistent condom use in the past month across the five-year window (Table 2).

**Table 2:** Demographic and behavioral characteristics of Vietnamese FSW by different years (2010–2014)

		2010 (N = 49)	2011 (N = 110)	2012(N = 166)	2013 (N = 343)	2014 (N = 358)	Overall (N = 1026)	p-value	p-trend
		% (n)	% (n)	% (n)	% (n)	% (n)	% (n)		
Age	*>25*	59.18 (29)	48.18 (53)	51.20 (85)	53.64 (184)	58.10 (208)	54.48 (559)	0.2970	0.1700
	*<=25 yrs*	40.82 (20)	51.82 (57)	48.80*81)	46.36 (159)	41.90 (150)	45.52 (467)		
Marital status	*Not married*	57.14 (28)	52.73 (58)	56.02 (93)	51.90 (178)	43.85 (157)	50.1 (514)	0.0470	0.0100
	*Married*	42.86 (21)	47.27 (52)	43.98 (73)	48.10 (165)	56.15 (201)	49.9 (512)		
Length of working	*<6 m*	18.37 (9)	64.55 (71)	39.63 (65)	56.85 (195)	48.60 (174)	50.2 (514)	0.0000	0.1560
	*> = 6 m*	81.63 (40)	35.45 (39)	60.37 (99)	42.15 (148)	51.40 (184)	49.80 (510)		
Education	*<MS*	61.22 (30)	56.36 (62)	54.88 (90)	41.69 (143)	53.91 (13)	50.59 (518)	0.0002	0.2150
	*> = MS*	38.78 (19)	43.64 (48)	45.12 (74)	58.31 (200)	46.09 (165)	49.41 (506)		
Community service	*Yes*	95.92 (47)	73.47 (72)	90.96 (151)	93.0 (319)	86.87 (311)	88.76 (900)	0.0000	0.4490
	*No*	4.08 (2)	26.53 (26)	9.04 (15)	7.00 (24)	13.13 (47)	11.24 (114)		
Condom use in the last sex	*No*	16.67 (8)	15.74 (17)	13.25 (22)	7.89 (27)	3.08 (11)	8.33 (85)	0.0000	0.0000
	*Yes*	83.33 (40)	84.26 (91)	86.75 (144)	92.11 (315)	96.92 (346)	91.67 (936)		
Condom use in the last month	*Inconsistent*	44.9 (22)	39.81 (43)	32.73 (54)	21.83 (74)	17.6 (63)	25.12 (256)	0.0000	0.0000
	*Consistent*	55.1 (27)	60.19 (65)	67.27 (111)	78.17 (265)	82.40 (295)	74.88 (763)		
Drug use	*No*	95.92 (47)	99.08 (108)	96.39 (160)	96.79 (332)	96.65 (346)	96.88 (993)	0.7170	0.6120
	*Yes*	4.08 (2)	0.92 (1)	3.61 (6)	3.21 (11)	3.35 (12)	3.12 (32)		
HIV knowledge	*<=5*	59.18 (29)	34.55 (38)	62.65 (104)	54.81 (188)	60.34 (216)	56.04 (575)	0.0000	0.0110
	*>5*	40.82 (20)	65.45 (72)	37.35 (62)	45.19 (155)	39.66 (142)	43.96 (451)		
Types of venues	*Low-paying*	83.67 (41)	72.48 (79)	73.49 (122)	50.00 (161)	48.74 (174)	57.33 (577)	0.0000	0.0000
	*Medium-paying*	6.12 (3)	17.43 (19)	19.28 (32)	43.48 (140)	50.70 (181)	37.39 (375)		
	*High-paying*	10.20 (5)	10.09 (11)	7.23 (12)	6.52 (21)	0.56 (2)	5.08 (51)		
Prevalence (%,95 % CI)	*HIV*	8.20 % (0.52 %,15.88 %)	5.50 % (1.24 %,9.76 %)	3.60 % (1.34 %,5.06 %)	3.20 % (1.34 %,5.06 %)	1.70 % (0.36 %,3.04 %)	3.22 % (2.12 %,4.28 %)	0.0770	0.0050
	*Syphilis*	8.20 % (0.52 %,15.88 %)	9.10 % (3.73,14.47 %)	9.60 % (5.12 %,14.08 %)	7.90 % (5.05 %,10.75 %)	3.90 % (1.89 %,5.91 %)	6.92 % 95.35 %,8.45 %)	0.0800	0.0210
	*HCV*	0.00 % (0.00 %,0.00 %)	2.70 % (−0.33 %,5.73 %)	2.40 % (0.07 %,4.73 %)	3.50 % (1.56 %,5.44 %)	2.20 % (0.68 %,3.72 %)	2.63 % (1.63 %,.57 %)	0.6290	0.6660

### Prevalence of infections in relation to demographic and behavioral factors

In Table 3, 4 and 5, we examined the prevalence of different types of infection across different strata of demographic and behavioral factors over time. HIV prevalence changed notably among FSW who were married and younger than 25 years old, stayed more than six months, had less education, reported no drug use, and those who participated in the HIV prevention services (*P* < 0.05; Table 3). Syphilis remained relatively stable among FSW who reported inconsistent condom use with clients in the past month, not participate in HIV prevention services, and lower HIV knowledge (*P* > 0.05; Table 4). No significant changes have been observed for HCV across the stratum over time (*P* > 0.05; Table 5).

**Table 3:** HIV prevalence by different behavioral and demographic characteristics among Vietnamese FSW by different years (2010–2014) (N = 1,026)

		2010	2011	2012	2013	2014	Overall	p-value	p-trend
		% (n/N)	% (n/N)	% (n/N)	% (n/N)	% (n/N)	% (n/N)		
Age	*>25*	6.9 (2/29)	5.66 (3/53)	3.53 (3/85)	4.35 (8/184)	2.40 (5/208)	3.76 (21/559)	0.633	0.154
	*<=25 yr*	10.0 (2/20)	5.26 (3/57)	3.70 (3/81)	1.89 (3/189)	0.67 (1/150)	2.57 (12/467)	0.063	0.005
Marital status	*Not married*	7.14 (2/28)	1.72 (1/58)	3.23 (3/93)	2.81 (5/178)	1.91 (3/157)	2.72 (14/514)	0.597	0.309
	*Married*	9.52 (2/21)	9.62 (5/52)	4.11 (3/73)	3.64 (6/165)	1.49 (3/201)	3.71 (19/512)	0.043	0.004
Length of working	*<6 m*	0 (0/9)	4.23 (3/71)	1.54 (1/65)	1.03 (2/195)	1.15 (2/174)	1.56 (8/514)	0.407	0.191
	*> = 6 m*	10.00 (4/40)	7.69 (3/39)	5.05 (5/99)	6.08 (9/148)	2.17 (4/184)	4.90 (25/510)	0.18	0.027
Education	*<MS*	13.33 (4/30)	6.45 (4/62)	3.33 (3/90)	5.59 (8/143)	2.59 (5/193)	4.63 (24/518)	0.089	0.024
	*> = MS*	0 (0/19)	4.17 (2/28)	4.05 (3/74)	1.50 (3/200)	0.61 (1/165)	1.78 (9/506)	0.24	0.119
Community service	*Yes*	8.51 (4/47)	5.56 (4/72)	3.97 (6/151)	3.13 (10/319)	1.93 (6/311)	3.33 (30/900)	0.128	0.006
	*No*	0 (0/2)	7.69 (2/26)	0 (0/15)	4.17 (1/24)	0 (0/47)	2.63 (3/114)	0.337	0.133
Condom use in the last sex	*No*	0 (0/8)	5.88 (1/17)	4.55 (1/22)	7.41 (2/27)	0 (0/11)	4.71 (4/85)	0.839	0.912
	*Yes*	0 (0/40)	3.30 (3/91)	2.08 (3/144)	3.49 (11/3150	2.31 (8/346)	2.67 (25/936)	0.66	0.761
Condom use in the last month	*Inconsistent*	0 (0/22)	2.33 (1/43)	5.56 (3/54)	6.76 (5/74)	0 (0/63)	3.52 (9/256)	0.183	0.994
	*Consistent*	7.41 (2/27)	3.08 (2/65)	3.60 (4/111)	3.77 (10/265)	1.69 (5/295)	3.01 (23/763)	0.38	0.115
Drug use	*Yes*	0 (0/2)	0 (0/1)	0 (0/6)	18.18 (2/22)	8.33 (1/12)	9.38 (3/32)	0.745	0.503
	*No*	8.51 (4/47)	5.56 (6/108)	3.75 (6/160)	2.71 (9/332)	1.45 (5/346)	3.02 (30/993)	0.032	0.002
Types of venues	*Low-paying*	7.32 (3/41)	7.59 (6/79)	3.28 (4/122)	5.59 (9/161)	2.87 (5/174)	4.68 (27/577)	0.373	0.112
	*Medium-paying*	0.00 (0/3)	0.00 (0/19)	6.25 (2/32)	1.43 (2/140)	0.55 (1/181)	1.33 (5/375)	0.135	0.267
	*High-paying*	20.00 (1/5)	0.00 (0/11)	0.00 (0/12)	0.00 (0/21)	0.00 (0/2)	1.96 (1/51)	0.052	0.063
HIV knowledge	*<=5*	10.34 (3/29)	7.89 (3/38)	4.81 (5/104)	3.19 (6/188)	1.85 (4/216)	3.65 (21/575)	0.087	0.003
	*>5*	5.00 (1/20)	4.17 (3/72)	1.61 (1/62)	3.23 (5/155)	1.41 (2/142)	2.66 (12/451)	0.669	0.234

**Table 4:** Syphilis prevalence by different behavioral and demographic characteristics among Vietnamese FSW by different years (2010–2014) (N = 1026)

		2010	2011	2012	2013	2014	overall	p-value	p-trend
		% (n/N)	% (n/N)	% (n/N)	% (n/N)	% (n/N)	% (n/N)		
Age	*<=25 yr*	10.0 (2/20)	3.51 (/57)	1.23 (1/81)	1.26 (2/159)	1.33 (2/150)	1.93 (9/467)	0.074	0.046
	*>25*	6.9 % (2/29)	15.09 (8/53)	17.65 (15/85)	13.59 (25/184)	5.77 (12/208)	11.09 (62/559)	0.016	0.045
Marital status	*Not married*	10.71 (3/28)	6.90 (4/58)	3.23 (3/93)	3.93 (7/178)	1.27 (2/157)	3.70 (19/514)	0.084	0.008
	*Married*	4.76 (1/21)	11.54 (6/52)	17.81 (13/73)	12.12 (20/165)	5.97 (12/201)	10.16 (52/512)	0.04	0.138
Length of working	*<6 m*	11.11 (1/9)	5.63 (4/71)	4.62 (3/65)	2.56 (5/195)	1.15 (2/174)	2.92 (15/514)	0.156	0.016
	*> = 6 m*	7.50 (3/40)	15.38 (6/39)	12.12 (12/99)	14.86 (22/148)	6.52 (12/184)	10.78 (55/510)	0.111	0.312
Education	*<MS*	10.0 (3/30)	12.9 (8/62)	11.11 (10/90)	15.38 (22/143)	3.63 (7/193)	9.65 (50/518)	0.006	0.03
	*> = MS*	5.26 (1/19)	4.17 (2/48)	8.11 (6/74)	2.50 (5/200)	4.24 (7/165)	4.15 (21/506)	0.361	0.475
Community service	*Yes*	8.51 (4/47)	11.11 (8/72)	9.27 (14/151)	8.15 (26/319)	3.86 (12/311)	7.11 (64/900)	0.076	0.01
	*No*	0.00 (0/2)	7.69 (2/26)	13.33 (2/15)	4.17 (1/24)	4.26 (2/47)	6.14 (7/114)	0.729	0.467
Condom use in the last sex	*No*	12.50 (1/8)	11.76 (2/17)	13.64 (3/22)	29.63 (8/27)	0 (0/11)	16.47 (14/85)	0.195	0.726
	*Yes*	7.5 (3/40)	8.79 (8/91)	9.03 (13/144)	6.03 (19/315)	4.05 (14/346)	6.09 (57/936)	0.199	0.032
Condom use in the last month	*Inconsistent*	9.09 (2/22)	11.63 (5/43)	16.67 (9/54)	21.62 (16/74)	6.35 (4/63)	14.06 (36/256)	0.109	0.9
	*Consistent*	7.41 (2/27)	7.69 (5/65)	6.31 (7/111)	3.77 (10/265)	3.39 (10/295)	4.46 (34/763)	0.39	0.057
Drug use	*Yes*	0 (0/2)	0 (0/1)	16.67 (1/6)	72.73 (8/11)	16.67 (2/12)	34.38 (11/32)	0.024	0.559
	*No*	8.51 (4/57)	9.26 (10/108)	9.38 (15/160)	5.72 (19/332)	3.47 (12/346)	6.04 (60/993)	0.046	0.006
Types of venues	*Low-paying*	9.76 (4/41)	10.13 (8/79)	12.30 (15/122)	12.42 (20/161)	5.17 (9/174)	9.71 (56/577)	0.172	0.153
	*Medium-paying*	0.00 (0/3)	5.26 (1/19)	3.13 (1/32)	5.00 (7/140)	2.76 (5/181)	3.73 (14/375)	0.849	0.616
	*High-paying*	0.00 (0/5)	0.00 (0/11)	0.00 (0/12)	0.00 (0/21)	0.00 (0/2)	0.00 (0/51)	n/a	n/a
HIV knowledge	*Low < =5*	6.90 (2/29)	7.89 (3/38)	7.65 (9/104)	9.04 (17/188)	4.63 (10/216)	7.13 (41/575)	0.481	0.266
	*High >5*	10.0 (2/20)	9.72 (7/72)	11.29 (7/62)	6.45 (10/155)	2.82 (4/142)	6.65 (4/142)	0.137	0.016

**Table 5:** HCV prevalence by different behavioral and demographic characteristics among Vietnamese FSW by different years (2010–2014) (N = 1026)

		2010	2011	2012	2013	2014	overall	p-value	p-trend
		% (n/N)	% (n/N)	% (n/N)	% (n/N)	% (n/N)	% (n/N)		
Age	*<=25 yr*	0 (0/29)	5.66 (3/53)	3.53 (3/85)	5.98 (11/184)	3.37 (7/208)	4.29 (24/559)	0.498	0.849
	*>25 = 1*	0 (0/20)	0 (0/57)	1.23 (1/81)	0.63 (1/159)	0.67 (1/150)	0.64 (3/467)	0.918	0.73
Marital status	*Not Married*	0 (0/28)	0 (0/58)	1.08 (1/93)	2.81 (5/178)	1.27 (2/157)	1.56 (8/514)	0.493	0.327
	*Married*	0 (0/21)	5.77 (3/52)	4.11 (3/73)	4.24 (7/165)	2.99 (6/201)	3.71 (19/512)	0.757	0.761
Length of working	*<6 m*	0 (0/9)	0 (0/71)	0 (0/65)	2.56 (5/195)	1.72 (3/174)	1.56 (8/514)	0.46	0.209
	*> = 6 m*	0 (0/40)	7.69 (3/39)	4.04 (4/99)	4.73 (7/148)	2.72 (5/184)	3.73 (19/510)	0.377	0.884
Education	*<MS*	0 (0/30)	1.61 (1/62)	3.33 (3/90)	6.29 (9/143)	3.11 (6/193)	3.67 (19/518)	0.301	0.317
	*> = MS*	0 (0/19)	4.17 (2/48)	1.35 (1/74)	1.50 (3/200)	1.21 (2/165)	1.58 (8/506)	0.637	0.499
Community service	*Yes*	0 (0/47)	2.78 (2/72)	2.65 (4/151)	3.45 (11/319)	2.25 (7/311)	2.67 (24/900)	0.69	0.657
	*No*	0 (0/2)	3.85 (1/26)	0 (0/15)	4.17 (1/24)	2.13 (1/47)	2.63 (3/114)	0.928	0.886
Condom use in the last sex	*No*	0 (0/8)	0 (0/17)	4.55 (1/22)	3.70 (1/27)	0 (0/11)	2.35 (2/85)	0.819	0.695
	*Yes*	0 (0/40)	3.30 (3/91)	2.08 (3/144)	3.49 (11/315)	2.31 (8/346)	2.67 (25/936)	0.66	0.761
Condom use in the last month	*Inconsistent*	0 (0/22)	2.33 (1/43)	5.56 (3/54)	6.76 (5/74)	0 (0/63)	3.52 (9/256)	0.183	0.994
	*Consistent*	0 (0/27)	3.08 (2/65)	0.90 (1/111)	2.26 (6/265)	2.71 (8/295)	2.23 (17/763)	0.727	0.414
Drug use	*Yes*	0 (0/2)	100.0 (1/1)	33.33 (2/6)	63.64 (7/11)	33.33 (4/12)	43.75 (14/32)	0.248	0.772
	*No*	0 (0/47)	1.85 (2/108)	1.25 (2/160)	1.51 (5/332)	1.16 (4/346)	1.31 (13/993)	0.904	0.077
Types of venues	*Low-paying*	0.00 (0/41)	3.80 (3/79)	2.46 (3/122)	4.35 (7/161)	3.45 (6/174)	3.29 (19/577)	0.681	0.93
	*Medium-paying*	0.00 (0/3)	0.00 (0/19)	3.13 (1/32)	2.86 (1/32)	1.10 (2/181)	1.87 (7/375)	0.732	0.716
	*High-paying*	0.00 (0/5)	0.00 (0/11)	0.00 (0/11)	0.00 (0/21)	0.00 (0/2)	0.00 (0/51)	n/a	n/a
HIV knowledge	*Low < =5*	0 (0/29)	5.26 (2/38)	2.88 (3/104)	4.26 (8/188)	3.24 (7/216)	3.48 (20/575)	0.756	0.705
	*High > 5*	0 (0/20)	1.39 (1/72)	1.61 (1/62)	2.58 (4/155)	0.70 (1/142)	1.55 (7/451)	0.723	0.974

### Predictive models for risk factors of different types of infection

In the predictive model using HIV as the outcome variable, univariate analyses revealed higher education, shorter stay in the current city, infection of syphilis and HCV, older age, drug use and working in low-paying venues were risk factors for HIV infection. Multivariate regression analyses revealed that infection of HCV marginally increased the risk of HIV infection by five-fold (aOR = 4.14, 95 % CI = 0.89, 19.36).

Where syphilis served as the outcome variable in the predictive model, univariate analyses found every covariate except for the HIV knowledge having significant associations. In the multivariate model, younger age (aOR = 1.18, 95 % CI = 1.01, 1.37), inconsistent condom use in the last sex act (aOR = 2.27, 95 % CI = 1.22-4.22), and infection of HCV (aOR = 4.78, 95 % CI = 1.53, 14.90) were risk factors for syphilis infection. For HCV, infection with syphilis (aOR = 3.64, 95 % CI = 1.20, 11.00) and drug use (aOR = 44.01, 95 % CI = 16.34, 129.53) significantly increased the odds of having HCV infection in the final model. All models showed good predictive ability with the AUC values ranged from 0.80 (95 % CI = 0.75, 0.84), to 0.84 (95 % CI = 0.80, 0.88) (Table 6).

**Table 6:** Predictive models for each different type of infection as outcome variables (N = 1026)^1^

	HIV		Syphilis		HCV	
PREDICTORS	Univariate	Multivariable^a^	Univariate	Multivariable^b^	Univariate	Multivariable^c,#^
	OR (95 % CI)	aOR (95 % CI)^4^	OR (95 % CI)	aOR (95 % CI)^4^	OR (95 % CI)	aOR (95 % CI)^4^
Education	2.68 (1.23,5.83)***	1.82 (0.79,4.20)	2.47 (1.46,4.17)****	0.92 (0.49,1.73)	2.37 (1.03,5.46)***	1.68 (0.59,4.77)
Length of working	0.31 (0.14,0.69)****	0.51 (0.21,1.25)	0.25 (0.14,0.45)******	0.60 (0.30,1.18)	0.41 (0.18,0.95)***	1.53 (0.50,4.67)
Marital status	0.73 (0.36,1.47)	N/A	0.34 (0.20,0.58)******	0.96 (0.49,1.91)	0.41 (0.18,0.95)***	0.78 (0.27,2.26)
No condom use in the last sex act	1.60 (0.55,4.68)	N/A	3.04 (1.62,5.72)****	1.55 (0.68,3.53)	0.88 (0.20,3.77)	N/A
Inconsistent condom use in the past month	1.31 (0.61,2.79)	N/A	3.51 (2.14,5.74)******	2.27 (1.22,4.22)***	1.60 (0.70.3.63)	N/A
HIV knowledge	0.82 (0.46,1.45)	N/A	0.84 (0.56,1.25)	N/A	0.58 (0.30,1.13)	0.63 (0.27,1.48)
Syphilis infection	3.17 (1.26,7.96)***	1.58 (0.56,4.46)	N/A	N/A	9.05 (3.97,20.59)******	3.64 (1.20,11.00)***
HCV infection	5.82 (1.89.17.90)****	4.14 (0.89,19.36) **	9.05 (3.97,20.60)******	4.78 (1.53,14.90)****	N/A	N/A
Age (continuous)^2^	1.05 (1.01,1.08)****	1.01 (0.98,1.05)	1.09 (1.06,1.11)******	N/A	1.05 (1.01,1.09)****	1.01 (0.95,1.07)
Age-younger (RCS)^3^	N/A	N/A	N/A	1.18 (1.01,1.37)***	N/A	N/A
Age-older (RCS)^3^	N/A	N/A	N/A	0.90 (0.76,1.07)	N/A	N/A
Drug use	3.32 (0.96, 11.51)*	0.84 (0.15,4.82)	8.15 (3.75,17.68)******	2.56 (0.87,7.49)**	58.63 (24.14,142.39)******	44.01 (16.34,129.53)******
Venues types (medium vs. low)	0.28 (0.11,0.72)***	0.46 (0.17,1.28)	0.36 (0.20,0.66)****	0.83 (0.41,1.65)	0.56 (0.23,1.34)	0.34 (0.09,1.38)
Venues types (high vs. low)	0.41 (0.05,3.06)	0.91 (0.11,7.46)	N/A	N/A	N/A	N/A

Notes: 1.All predictive models employed multiple imputation to deal with missing values; 2. LRT is non-significant for the RCS terms; 3.RCS-Restricted Cubic Spline with 3 knots for age; 4. aOR (95 % CI): adjusted odds ratios and its 95 % confidence intervals.

Model a’s AUC is 0.84 (0.80-0.88), Model b’ AUC is 0.80 (0.75-0.84), Model c’s AUC is 0.82 (0.70, 0.93). Areas under the ROC curve (AUC) are used to provide the predictive value for each model.

#-this model employed MI to impute missing values of HIV knowledge, as the missing values have more than 20 %

*p < 0.20, **0.05 < p < 0.10, ***p < 0.05, ****p < 0.005, ******p < 0.0001

## Discussion

In the current study, we used serial cross-sectional NSS surveys to analyze the epidemiology of the HIV, syphilis and HCV epidemic among cross-border Vietnamese FSW in Guangxi province of China. The data showed that both the absolute number and relative percentages of Vietnamese FSW rose quickly over time, ranging from 49 (1.09 %) in 2010, 109 (1.60 %) in 2011, 166 (1.97 %) in 2012, 322 (3.02 %) in 2013, to 357 (3.64 %) in 2014. The ever-increasing number of Vietnamese FSW may be due to the rapid economic development in China, where many Vietnamese unskilled women come to seek for working opportunities in China, but end up entering the sex industry. The rampant criminal sex trafficking at the Vietnam-China border may exacerbate the situation [34]. The recent financial crisis and economic recession of the United States and other major trade partners of Vietnam worsened the already fragile market in Vietnam [35]. Therefore, many Vietnamese have to look for financial survival outside their home country, and mainland China is one of their destinations. For women with low skills or who are desperate for quick financial return, they are very likely to end up being involved in sex industry [36].

Compared to other large-scale FSW studies conducted in China, 3–10 times higher HIV prevalence was observed among Vietnamese FSW in the current study even after standardization [37]. The higher risk of cross-border FSW may be because of the lack of responsive capacities to local HIV prevention strategies due to language and cultural barriers [7, 10], or the lack of optimal methods specifically targeting Vietnamese FSW, such as the VCT or rapid testing programs [10]. The pattern of increased condom use among this group was consistent with the pattern among the Chinese FSW [38]. Probably the intensive outreach programs of promoting condom use in the recent years contributed to the increasing trend [39]. Meanwhile, due to the venue-based sampling strategy, most FSW in the NSS data were recruited from brothel-based settings, where managers/gatekeepers had strong motivation of maintaining the health of their FSW in order to keep a steady and healthy workforce [36]. Future studies need also to include Vietnamese FSW worked as free-lancers, as they may have much higher risk of contracting HIV and other sexually transmitted diseases (STD) [40, 41].

Although decreasing trends of both HIV and syphilis prevalence were detected, a relatively stabilizing HCV prevalence over time among the Vietnamese FSW in the current NSS sample. The decreasing trends could be due to the underestimation of HIV and syphilis prevalence, as a result of the small sample size at the initial stage of the evaluation (e.g., only 49 Vietnam FSW recruited in 2010). Similar to majority of the Chinese FSW, drug use were the main risk factors of contracting HCV [42, 43]. Among these drug users, needle sharing could be the main route of transmission. In addition, drug use may disinhibit risk perceptions and promote risky behaviors [42]. Furthermore, some women trapped by the vicious drug-sex circle featured by exchanging sex for drugs [12, 42].

The analysis of the serial cross-sectional NSS data has several strengths including large sample size across five continuous years, rigorous model building strategies as well as sensitivity analyses (e.g., multiple imputation and RCS), and all these strengths may increase the confidence of our estimations [30]. Based upon findings in the current study, HCV served as a key risk factor for infections of HIV and syphilis. More attention should be paid to HCV infection beyond the scope of existing HIV/syphilis prevention programs. In the existing healthcare infrastructure, many free or discounted medical services are only open to Chinese citizens for their HIV infection. With the exploding number of foreign FSW working in China and the steady HCV epidemic across the past five years, we call for mobilizing the local community and local government at the cross-border areas to pay more attention to this issue and to provide necessary and affordable medical services to this population with their STD treatment and drug use management. In addition, a special surveillance system can be set up for this particular population. Once a case (not only HIV, but also HCV or syphilis) has been identified, the data should be reported to both sides. With the open and transparent reporting system, governments at both sides can implement effective programs in a timely manner.

A few limitations should be considered when interpreting our findings. First, no casual inference can be drawn based upon these serial cross-sectional surveys. Second, because of the space constraint in the survey, some key covariates that may be significantly associated with HIV risks among participants were not collected [36, 40, 42–45]. Third, the social desirability is always a concern for sensitive topics, and these biases may result in underreporting risky behaviors of the respondents. Fourth, as all data in the current study were collected in Guangxi province, it may constrain its generalizability to other regions of China.

## Conclusion

In summary, an ever-increasing epidemic over the five-year window has been identified among the Vietnamese FSW at the cross-border region. Drug use was the main risk factor for HCV which further served as a risk factor for HIV and syphilis infection in the current sample. To curb the epidemic among these cross-border FSW, a dose of humility and effective approaches are urgently needed, which requires bilateral cooperation and action.
